# Efficacy and safety of mesenchymal stem cells therapy in COVID-19 patients: a systematic review and meta-analysis of randomized controlled trials

**DOI:** 10.1186/s12967-024-05358-6

**Published:** 2024-06-08

**Authors:** Wenming Lu, Longxiang Yan, Xingkun Tang, Xuesong Wang, Jing Du, Zhengwei Zou, Lincai Li, Junsong Ye, Lin Zhou

**Affiliations:** 1 Subcenter for Stem Cell Clinical Translation, First Affiliated Hospital of Gannan Medical University, Ganzhou, 341000 Jiangxi People’s Republic of China; 2 School of Rehabilitation Medicine, Gannan Medical University, GanZhou City, 341000 Jiangxi People’s Republic of China; 3 Ganzhou Key Laboratory of Stem Cell and Regenerative Medicine, Ganzhou, 341000 Jiangxi People’s Republic of China; 4 The First Clinical College of Gannan Medical University, Ganzhou, 341000 Jiangxi People’s Republic of China; 5 Key Laboratory of Prevention and Treatment of Cardiovascular and Cerebrovascular Diseases, Ministry of Education, Gannan Medical University, Ganzhou, 341000 Jiangxi People’s Republic of China; 6 Jiangxi Provincal Key Laboratory of Tissue Engineering, Gannan Medical University, Ganzhou, 341000 Jiangxi People’s Republic of China

**Keywords:** Mesenchymal stem cells, COVID-19, Efficacy, Safety, Meta-analysis

## Abstract

**Background:**

The coronavirus disease 2019 (COVID-19) has become a serious public health issue. In COVID-19 patients, the elevated levels of inflammatory cytokines lead to the manifestation of COVID-19 symptoms, such as lung tissue edema, lung diffusion dysfunction, acute respiratory distress syndrome (ARDS), secondary infection, and ultimately mortality. Mesenchymal stem cells (MSCs) exhibit anti-inflammatory and immunomodulatory properties, thus providing a potential treatment option for COVID-19. The number of clinical trials of MSCs for COVID-19 has been rising. However, the treatment protocols and therapeutic effects of MSCs for COVID-19 patients are inconsistent. This meta-analysis was performed to systematically determine the safety and efficacy of MSC infusion in COVID-19 patients.

**Methods:**

We conducted a comprehensive literature search from PubMed/Medline, Web of Science, EMBASE, and Cochrane Library up to 22 November 2023 to screen for eligible randomized controlled trials. Inclusion and exclusion criteria for searched literature were formulated according to the PICOS principle, followed by the use of literature quality assessment tools to assess the risk of bias. Finally, outcome measurements including therapeutic efficacy, clinical symptoms, and adverse events of each study were extracted for statistical analysis.

**Results:**

A total of 14 randomized controlled trials were collected. The results of enrolled studies demonstrated that patients with COVID-19 pneumonia who received MSC inoculation showed a decreased mortality compared with counterparts who received conventional treatment (RR: 0.76; 95% CI [0.60, 0.96]; *p* = 0.02). Reciprocally, MSC inoculation improved the clinical symptoms in patients (RR: 1.28; 95% CI [1.06, 1.55]; *p* = 0.009). In terms of immune biomarkers, MSC treatment inhibited inflammation responses in COVID-19 patients, as was indicated by the decreased levels of CRP and IL-6. Importantly, our results showed that no significant differences in the incidence of adverse reactions or serious adverse events were monitored in patients after MSC inoculation.

**Conclusion:**

This meta-analysis demonstrated that MSC inoculation is effective and safe in the treatment of patients with COVID-19 pneumonia. Without increasing the incidence of adverse events or serious adverse events, MSC treatment decreased patient mortality and inflammatory levels and improved the clinical symptoms in COVID-19 patients. However, large-cohort randomized controlled trials with expanded numbers of patients are required to further confirm our results.

**Supplementary Information:**

The online version contains supplementary material available at 10.1186/s12967-024-05358-6.

## Introduction

Coronavirus disease 2019 (COVID-19) first appeared in December 2019 in the city of Wuhan, China [1–3], and rapidly became a global pandemic [4–6]. Since then, patients with COVID-19 have been rising globally. As of August 2022, there have been over 59 million cumulative COVID-19 patients, and more than 6 million deaths worldwide [7, 8].

COVID-19, a viral infectious disease, is caused by severe acute respiratory syndrome coronavirus 2 (SARS-CoV-2) infection [9]. Coronaviruses, single-strand and positive-sense RNA genetic groups, induce both enteric and respiratory diseases in the human body [9, 10]. Generally, the clinical manifestations of the patient include fever, fatigue, and non-productive cough. Typically, patients with dyspnea present with respiratory distress [11]. Rapid progression to acute respiratory distress syndrome (ARDS), multiple organ damage, and perhaps death is observed in serious COVID-19 cases [12, 13]. As of right now, there is no effective antiviral medication for COVID-19, and symptomatic and supportive therapies are the mainstay. COVID-19 has a unique immunopathology termed cytokine storm syndrome (CSS) [14], which is formed by immune cells that emit excessive cytokines and set off a positive feedback loop [15–17]. For patients with COVID-19 pneumonia, CSS is highly correlated with severe and critical disease. It also contributes to forecasting the severity of the illness's course and offers potential therapeutic targets [18]. Therefore, it is of great urgency to develop safe and effective treatment strategies for COVID-19.

Mesenchymal stem cells (MSCs), derived from the mesoderm, can be isolated from a variety of tissues, including bone marrow (BM), adipocytes (AD), umbilical cord blood, umbilical cord (UC), menses blood, dental pulp, placenta, amniotic fluid, and even the brain, spleen, liver, kidney, lung, thymus, and pancreas [19–22]. In recent years, the immunomodulatory and regenerative capacity of MSC has provided us with a viable treatment option for several inflammatory diseases, such as rheumatoid arthritis, ulcerative colitis, Crohn's disease (CD), and graft versus host disease (GVHD) [23–26]. Pre-clinical research has shown that MSC infusion in mice with ARDS improved the lung microenvironment, inhibited the hyperactive immune system, prompted tissue repair, shielded alveolar epithelial cells, and prevented pulmonary fibrosis, all of which improved survival rate [27–31]. More importantly, clinical trials also have shown that MSC inoculation suppressed lung inflammation and improved the oxygenation index and the survival rate in patients with COVID-19 [32–45]. In conclusion, MSC transplantation is a promising strategy for COVID-19 treatment. Some previous studies have demonstrated the efficacy and safety of MSCs in the treatment of COVID-19 or ARDS. However, potential future directions such as the optimal cell type, cell dose, and route of infusion still need to be further elaborated [46–49]. Unfortunately, few of these analyses had specifically focused on the effects of different cell types and routes of transplantation in MSC treatment upon given randomized controlled trials (RCTs). Herein, we screened and extracted data about MSCs against COVID-19 in clinical RCTs and aimed to tightly assess their efficacy and safety of MSC transplantation for COVID-19. Moreover, we also conducted sensitivity analyses to explore sources of heterogeneity between studies. Collectively, our results might provide propositions for elucidating the potential therapeutic role of MSC in COVID-19 treatment.

## Methods

The detailed agreement of this systematic review is registered in the PROSPERO. The registration ID is CRD42023491775 (https://www.crd.york.ac.uk/PROSPERO/). The preferred reporting items for systematic reviews and meta-analyses (PRISMA) statement (http://www.prisma-statement.org/) were used to guide this systematic review (Additional file 3).

### Search strategies

Five databases (PubMed, Embase, the Web of Science, and the Cochrane Library) were systematically searched for eligible studies, from their inception dates to November 2023, with publishing in English. The search keywords used subject words and free words which were (COVID-19 or SARS-COV-2) AND (mesenchymal stromal cells). The detailed search strategy is presented in Additional file 2. Manual searches of the reference lists of included studies and narrative reviews were also performed. The search was limited to English papers. Unpublished papers were not included in this meta-analysis.

### Study selection

Two authors (Xing-kun Tang and Xue-song Wang) independently searched the literature and screened those that met the inclusion criteria by browsing the title, abstract, and keywords, ineligible studies were excluded. Any disagreements were resolved through discussion between the two authors, and if necessary, a third author was consulted for a decision.

### Inclusion criteria

This meta-analysis was carried out based on PICOS principles (population, intervention, comparison, outcomes, and study design), as shown below:

Population (P): Patients diagnosed with COVID-19 positive, regardless of country, region, age, sex, and race.

Intervention (I): The evaluated studies were intervened by MSC transplantation. There were no limitations to the time, duration, or dose of MSC therapy.

Comparison (C): The control group received a placebo or other treatment.

Outcomes (O): mortality; clinical improvement rate; days to hospital stays; adverse events (AEs); serious adverse events (SAEs); the number of patients requiring respiratory support; duration of oxygen therapy; C-reactive protein (CRP); D-dimer levels; serum ferritin levels; procalcitonin (PCT); IL-10; fibrinogen.

Study design (S): Randomized controlled trials (RCTs) limited to English studies.

### Exclusion criteria

We excluded conference abstracts, letters with duplicates, case reports, meta-analyses, reviews, non-English published literature, and studies with incomplete or unavailable data. In addition, studies not relevant to this topic (such as studies using animal models and interventions that are not MSC transfusions) were excluded.

### Data extraction

The studies that we retrieved were managed using Endnote X9. All relevant data were extracted independently by two investigators (Wen-ming Lu and Long-xiang Yan) from the included studies using standardized data extraction forms in Microsoft Excel. The data were then summarized in Table 1. If a difference occurred, a third senior member of the team was consulted to ensure accuracy. We extracted data on the following: study characteristics including date of publication, first author, and study site. Patient characteristics include age, sex, COVID-19 severity, and sample size in the group (MSC and control group). Details of intervention involve MSCs dose, outcome, route of administration, and MSC type.

**Table 1:** The main characteristics of clinical randomized controlled trials of mesenchymal stem cell therapy in COVID-19 patients

Included studies	Country	COVID-19 severity	Design	Age(year)	Patients(con/exp)	Cell type	Cell dose	Administration route	Main outcome measures
Monse et al. [38]	French	Moderate, severe	RCT	>18	24, 21	UC-MSC	1 × 10^6^ cells/kg 3 times	Intravenous	Mortality, AE
Soetjahjo et al. [42]	Indonesia	Severe	RCT	18–75	21, 21	UC-MSC	1 × 10^6^ cells/kg	Intravenous	Duration of hospitalization, CRP levels, AE, SAE events
Meng et al. [37]	China	Moderate-severe	RCT	18–70	9, 9	UC-MSC	3 × 10^7^ cells/kg	Intravenous	CRP, Oxygen support, Clinical symptoms
Wei et al. [43]	China	Moderate, severe, critical	RCT	18-70	13, 12	UC-MSC	1 × 10^6^ cells/kg	Intravenous	Biomarker levels, AE, SAE events
Adas et al. [32]	Turkey	Critical	RCT	40-60	10,10	Wharton’s Jelly	3 × 10^6^ cells/kg 3 times	Intravenous	Mortality, AE, SAE, CRP, IL-6, IFN-γ
Lanzoni et al. [36]	America	Severe	RCT	>18	12, 12	UC-MSC	2 × 10^6^cells/kg	Intravenous	Mortality, AE, SAE, IL-6, IFN-γ
Dilogo et al [34]	Indonesia	Critical	RCT	18–95	20, 20	UC-MSC	1 × 10^6^ cells/kg	Intravenous	Mortality, AE, SAE
Shi et al. [40]	China	Critical	RCT	>18	35, 65	UC-MSC	8 × 10^5^ cells/kg	Intravenous	Mortality, AE, SAE, IL-6, IFN-γ
Shu et al. [41]	China	Severe	RCT	18–90	29, 12	UC-MSC	2 × 10^6^ cells/kg	Intravenous	Mortality, CRP, IL-6, days to hospital discharge
Bowdish et al. [33]	America	Moderate, severe	RCT	>18	110, 111	UC-MSC	2 × 10^6^ cells/kg 8 times	Intravenous	Mortality, AE, SAE, clinical improvement rate
Kaffash Farkhad et al. [35]	Iran	Non-severe	RCT	>18	10,10	UC-MSC	1 × 10^6^ cells/kg 3 times	Intravenous	Mortality, IL-6, CRP
Rebelatto et al. [39]	Brazil	Critical	RCT	>18	6, 11	UC-MSC	5 × 10^5^cells/kg 3 times	Intravenous	Mortality, IL-6, CRP, AE
Zhu et al. [44]	China	Moderate, severe, critical	RCT	18–95	29, 29	UC-MSC	1 × 10^6^ cells/kg	Intravenous	Mortality, clinical improvement rate, AE, days to hospital discharge
Xu et al. [29, 45]	China	Severe and critically	RCT	18–75	18, 26	Menstrual blood-MSC	6 × 10^5^ cells/kg	Intravenous	Time to improve, the number of days in hospital, stay in ICU

Con: control group; Exp: experimental group; COVID-19: Coronavirus disease 2019; RCT: randomized controlled trial; UC-MSCs: umbilical cord mesenchymal stem cells; CRP: C-reactive protein; TNF-α: tumor necrosis factor-alpha; IFN-γ: interferon-gamma; IL-1: interleukin-1; IL-6: interleukin-6; IL-2: interleukin-2; AEs, adverse events; SAEs, serious adverse events

### Assessment of the risk of *Bias* in the included studies

Two researchers used Review Manager (version 5.4) to assess the Risk of Bias based on the recommendations of the Cochrane Collaboration. Any discrepancies were resolved by consulting the third author. The risk of bias assessment form includes the following items: (1) random sequence generation (selection bias); (2) allocation concealment (selection bias); (3) blinding of participants and personnel (performance bias); (4) blinding of outcome assessment (detection bias); (5) incomplete outcome data (attrition bias); (6) selective reporting (reporting bias); and (7) other bias. Each dimension was classified as low risk, high risk, or unclear risk.

### Date analysis

This meta-analysis was conducted using Review Manager 5.4. Results for continuous outcomes evaluated in this article were expressed as the mean ± standard deviation (SD). The medians and interquartile ranges were converted into means and SD according to the conversion tools for subsequent analyses [50]. For dichotomous outcomes, relative risk (RR) with a 95% confidence interval (CI) was used to present the results [51]. For continuous variables, the weighted mean difference (WMD) and standardized mean difference (SMD) were used to compare the results. We used the chi-squared (χ2) statistical test and the inconsistency index (Ι^2^) statistic to assess the heterogeneity between studies. A value of *p* < 0.05 and I^2^ > 50% was considered to have significant heterogeneity and a random model and its index SMD were used for the combined analysis. If not then, the fixed effects model and its effect size WMD were used [52]. When individual studies were removed, we also carried out sensitivity analyses to evaluate the impact of each RCT on the overall results. Finally, *p* < 0.05 was considered statistically significant for all analyses.

## Results

### Results of the search

**Fig. 1:**
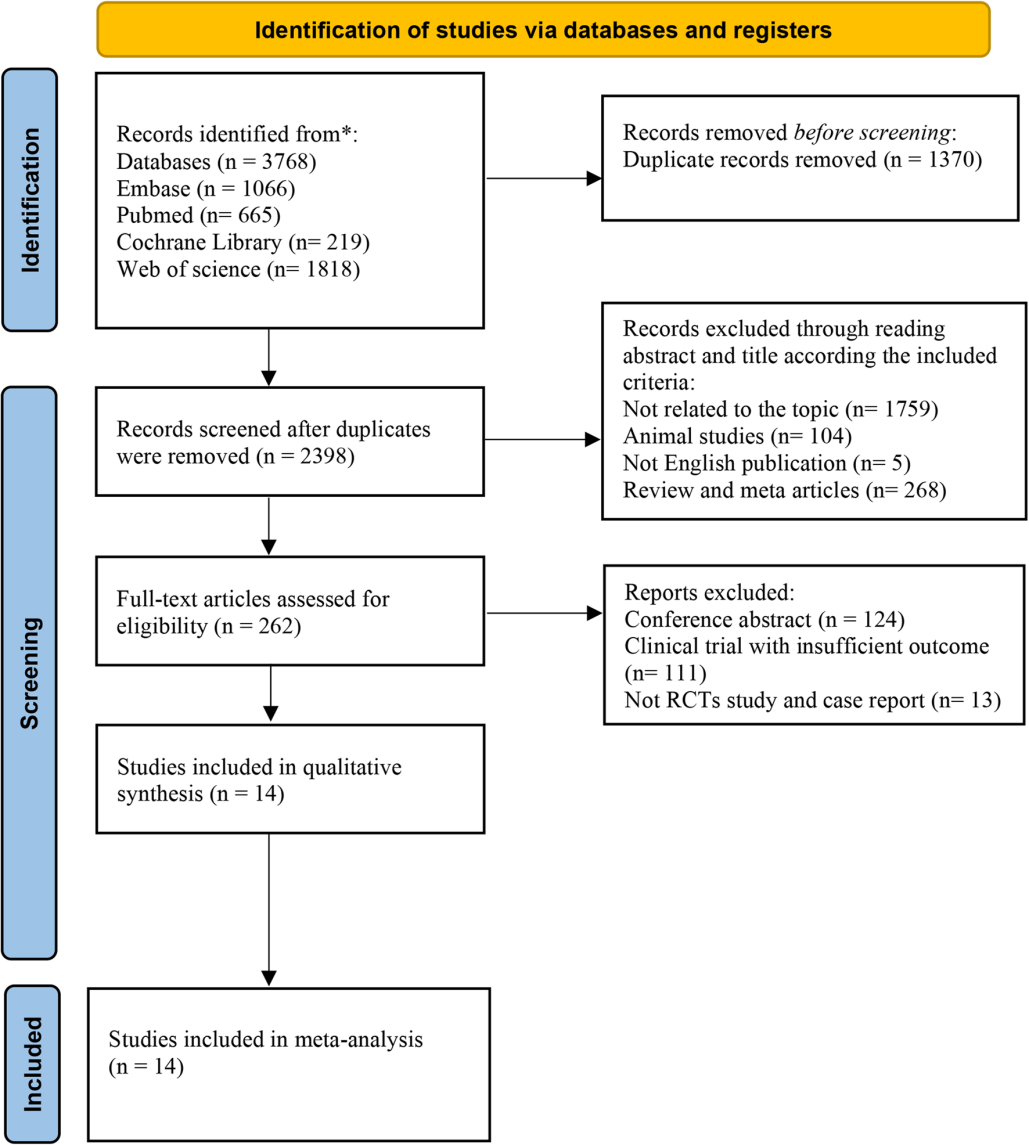
PRISMA 2020 flow diagram of the search strategy and study selection

In the initial search in databases, 3768 potentially eligible studies were finally retrieved from PubMed, Cochrane Library, Web of Science, and Embase databases. After removing duplicated studies, 2398 studies remained. Subsequently, the titles and abstracts of articles were read to the further screen; 2136 articles were excluded for the following reasons: they were either animal experimental models, with no relevant topics, not English publications, or previous reviews and meta. 262 studies were included for full-text review. After careful review, 248 articles were removed due to conference abstracts without full text and relevant data, clinical trials with insufficient outcomes, and non-RCTs. Eventually, 14 clinical studies consisting of 715 patients were included in this meta-analysis. Figure 1 shows details of the study selection process.

### Characteristics of the studies

Fourteen eligible RCTs were enrolled for meta-analysis. These studies were conducted in 7 countries: six studies recruited participants in China, 2 studies each came from America and Indonesia, and the remaining four studies were from France, Iran, Indonesia, and Brazil respectively. All patients were diagnosed with COVID-19 and were over 18 years old. The severity of COVID-19 patients is varied, including moderate, severe, and critical. 12 clinical trials used UC-MSC as a therapy cell, and 2 studies used separately Wharton’s Jelly and menstrual blood-MSC in the treatment group. MSC was infused in doses ranging from 5 × 10^5^ cells/kg to 3 × 10^7^ cells/kg per participant in included studies. The route of MSC infusion for all studies was intravenous. As for the outcome, most of the articles reported mortality results, AEs, SAEs, and immune biomarkers. The included studies were published ranging from 2020 to 2022. The baseline characteristics of the included studies are summarized in Table 1**.**

### Risk assessment of *bias*

**Fig. 2:**
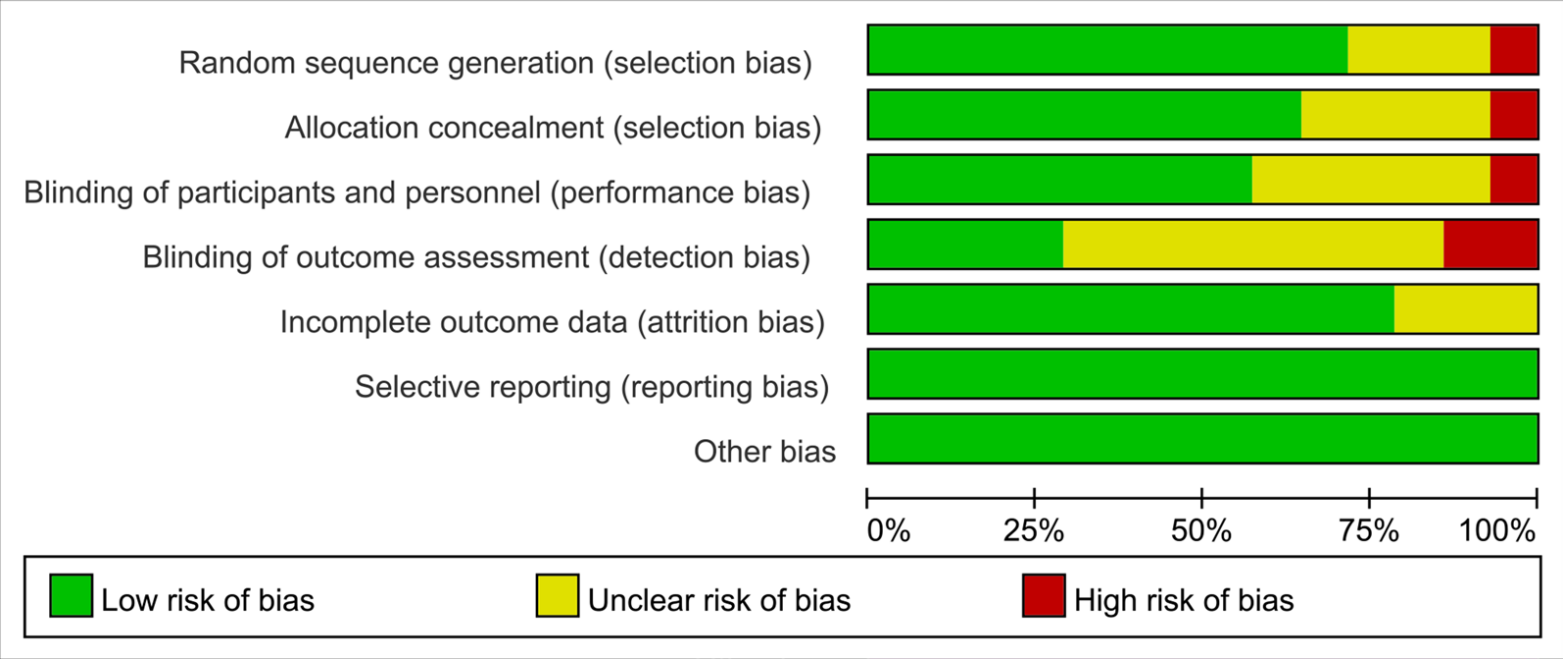
Risk of bias graph: review authors' judgments about each risk of bias item presented as percentages across all included studies

**Fig. 3:**
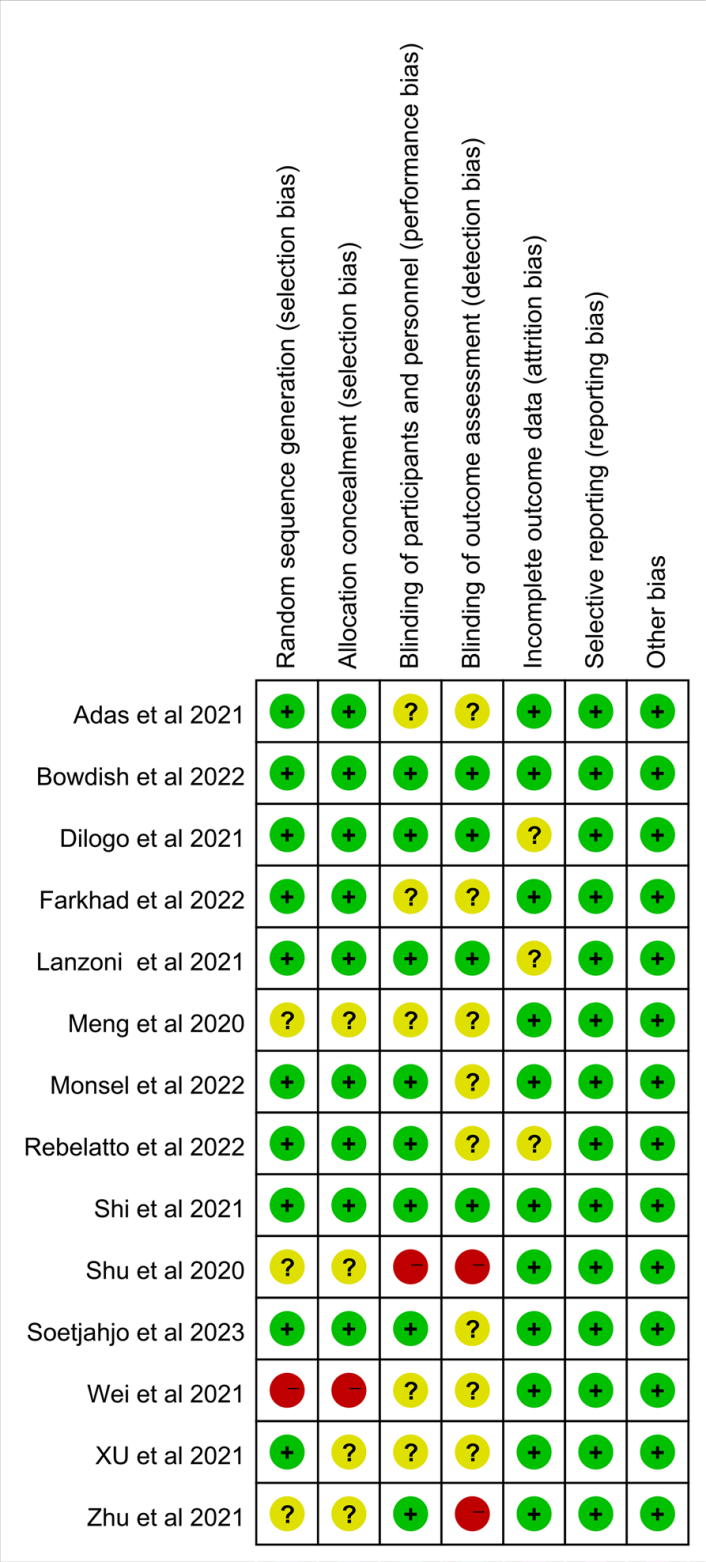
Risk of bias summary: review authors' judgments about each risk of bias item for each included study

The results of the quality assessment for each study are presented in Figs. 2 and 3**.** The green sign refers to a low risk of bias, the yellow sign refers to an unclear risk of bias, and the red sign refers to a high risk of bias. We observed that random outcome generation had a low risk of bias in ten studies, three studies did not mention it (unclear risk of bias), and one study was grouped according to patients' wishes (high risk of bias). For random outcome generation, nine studies were at low risk of bias, four were not reported (unclear risk of bias), and one study was at high risk of bias. Allocation concealment was mentioned in eight studies (low risk of bias) and was not mentioned in five studies (unclear risk of bias), one study had a high risk of bias. For the blinding of the outcome assessment, four retrieved studies were low risk, and ten were not mentioned (unclear risk of bias), two single-blind procedures were reported (high risk of bias). The majority of studies have complete information results (low risk of bias). All included studies had no selective reporting bias or other bias. The general risk of bias in the included studies was acceptable.

### *Meta*-analysis

Fourteen eligible studies were included for meta-analysis using a random-effects model, with mortality, AEs, and SAEs as primary and clinical improvement rates, days to hospital stays, the number of patients requiring respiratory support, duration of oxygen therapy and immune biomarkers as secondary indicators to evaluate the effectiveness of MSC for the COVID‐19 patients.

#### Primary indicators

##### Mortality

**Fig. 4:**
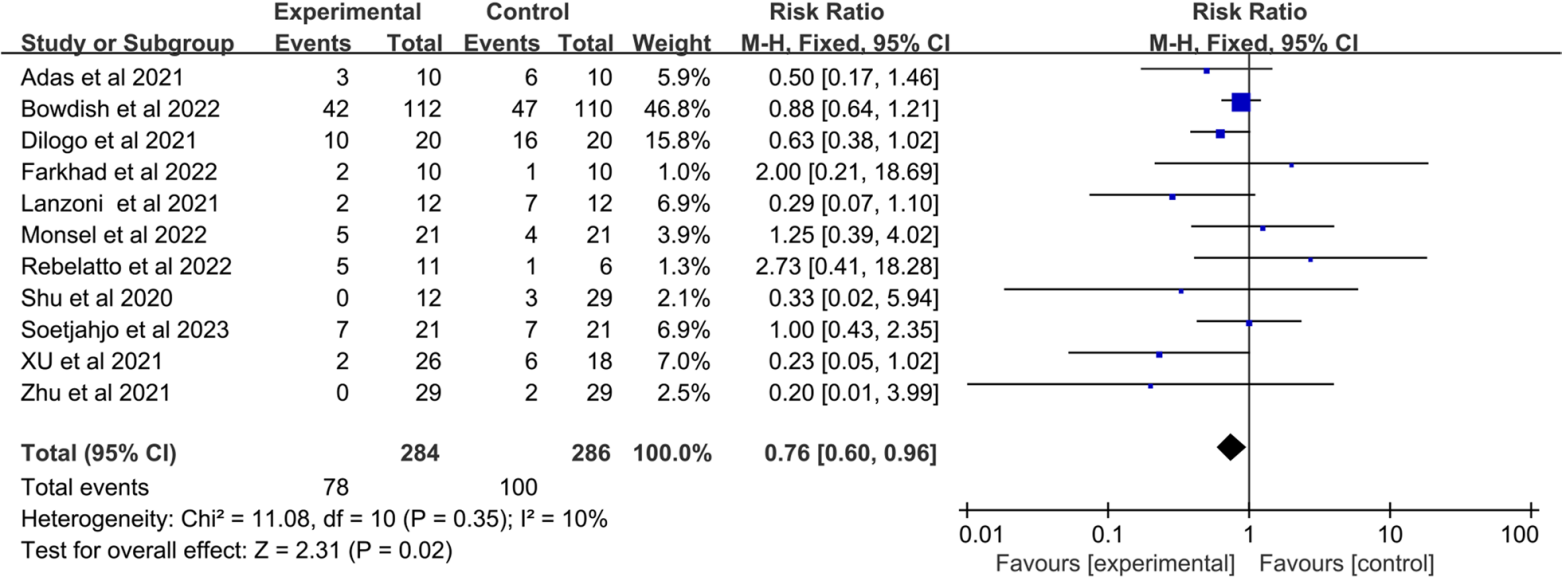
Forest plot of primary indicator: pooled results of mortality

The mortality was reported in eleven studies of 284 patients in the MSC group and 286 patients in the control group. The heterogeneity between studies was examined, and low heterogeneity was found (I^2^ = 10%; Q test *p* = 0.35). Therefore, the fixed effects model was adopted. The pooled data indicated a substantial difference between the two groups was observed (RR: 0.76; 95% CI [0.60, 0.96]; *p* = 0.02) (Fig. 4). The mortality rate was 0.76‐fold among individuals who received MSCs compared with patients who received conventional treatment. This finding suggested that COVID-19 patients receiving MSC treatment may have better survival rates.

##### AEs

**Fig. 5:**
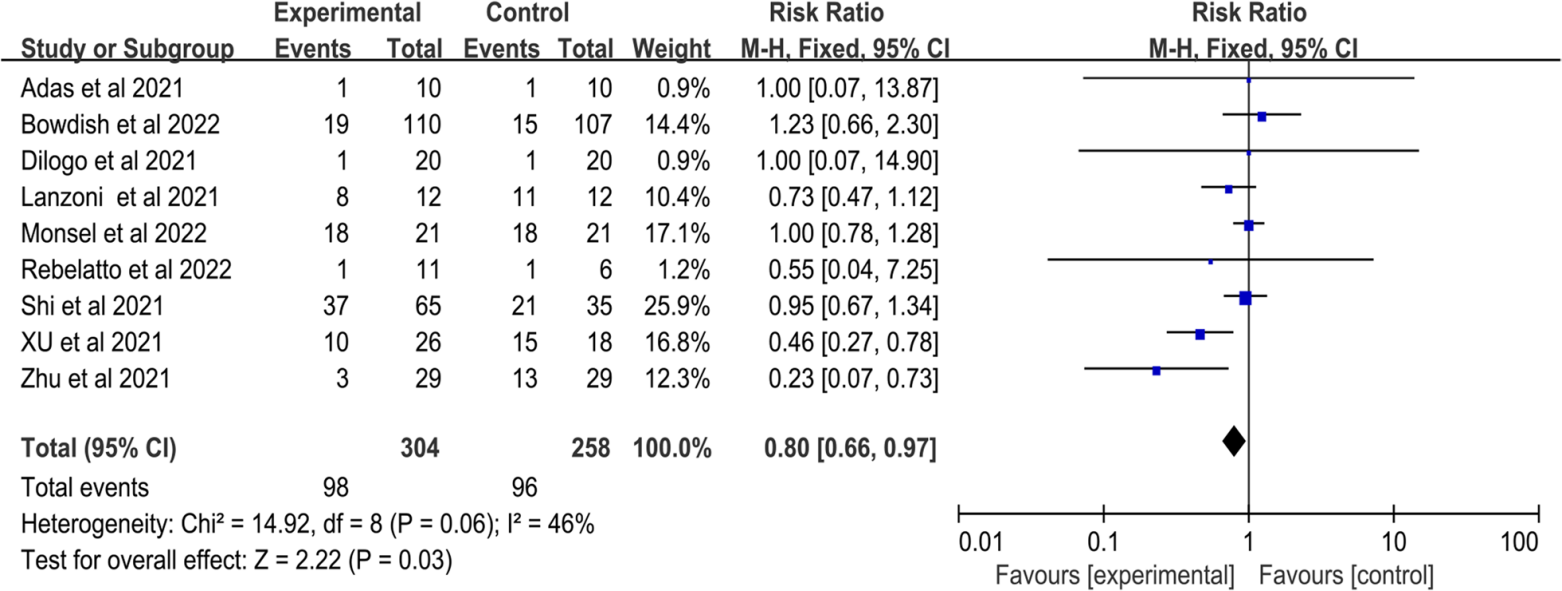
Forest plot of primary indicator: pooled results of AEs

The incidences of AEs were presented in nine RCTs involving 304 patients in the MSC group and 258 patients in the control group. A fix-effects meta-analysis indicated the MSC group was associated with significantly lower incidences of AEs than the control group (RR: 0.80; 95% CI [0.66, 0.97]; *p* = 0.03), suggesting infusion of MSC did not increase the occurrence of AEs. A moderate heterogeneity was observed among included studies (I^2^ = 46%; Q test *p* = 0.06) (Fig. 5).

##### SAEs

**Fig. 6:**
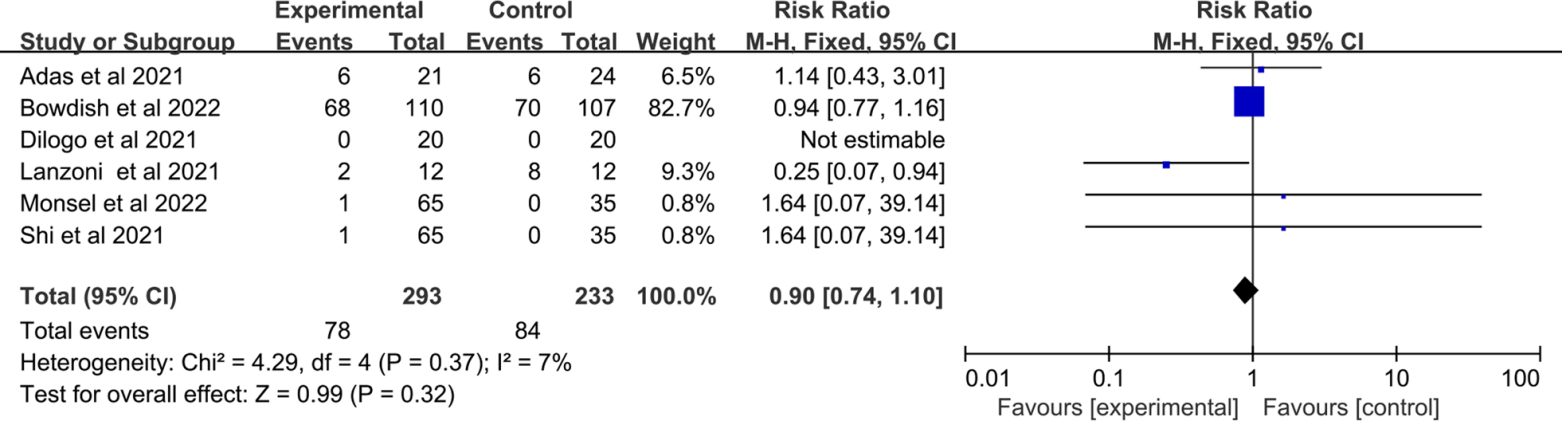
Forest plot of primary indicator: pooled results of SAEs

Among 14 studies included, SAEs were reported in six studies of 293 patients in the MSC group and 233 patients in the control group. Low heterogeneity was observed between the two groups (I^2^ = 7%; Q test *p* = 0.37). The fixed effects model was adopted. The pooling results suggested that no statistically significant differences were found in the occurrence of SAEs between the MSC group and the control group (RR: 0.90; 95% CI [0.74, 1.10]; *p* = 0.32) (Fig. 6). indicating that infusion of MSCs does not increase the number of SAEs with COVID‐19 patients.

#### Secondary indicators

##### Clinical improvement rate

**Fig. 7:**
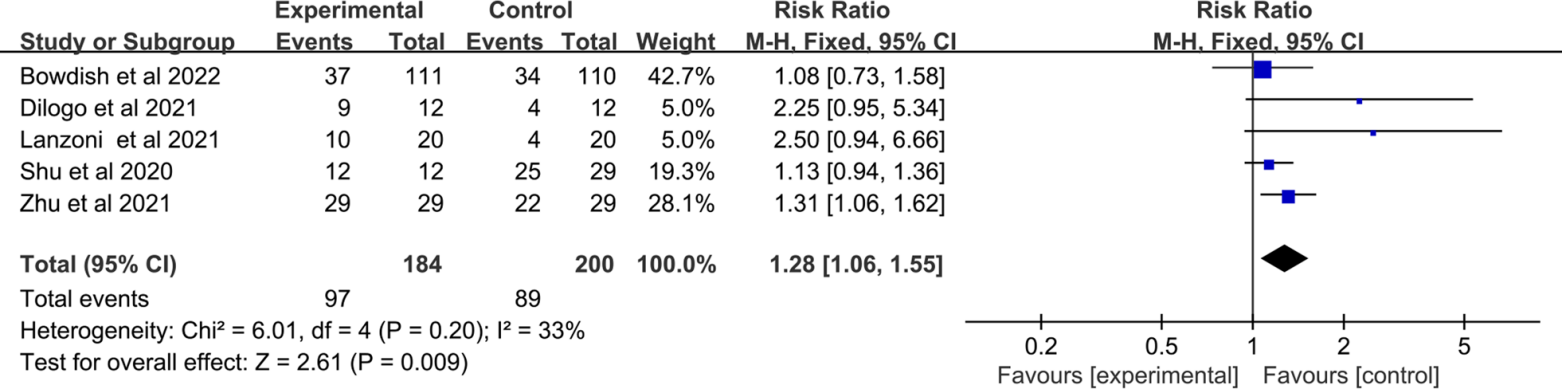
Forest plot of secondary indicators: clinical improvement rate

**Fig. 8:**
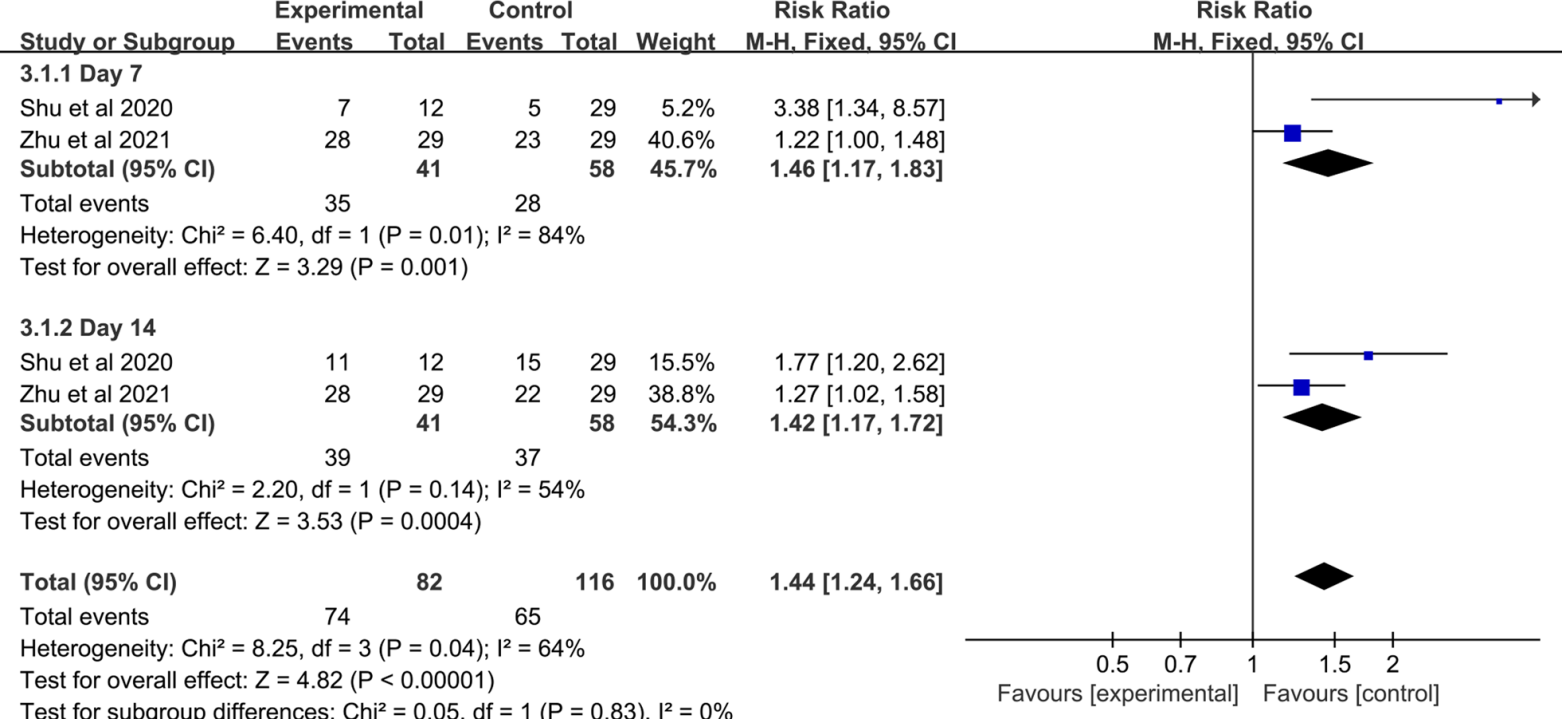
Forest plot of secondary indicators: time subgroup of clinical improvement rate

Five studies with a total of 384 patients were enrolled. The heterogeneity between studies was examined, and no heterogeneity was found (I^2^ = 33%; Q test *p* = 0.20). Therefore, the fixed effects model was implemented. The synthesized data showed that compared with control, MSC improved the clinical improvement rate in adult patients with COVID‐19 (RR: 1.28; 95% CI [1.06, 1.55]; *p* = 0.009) (Fig. 7). Furthermore, when the time subgroup analysis for clinical improvement rate was performed. Subgroup analysis with a fix-effects model demonstrated that the MSC group significantly increased clinical improvement rate in 7 days (RR: 1.46; 95% CI [1.17, 1.83]; *p* = 0.001; I^2^ = 84%), and 14 days (RR: 1.42; 95% CI [1.17, 1.72]; *p* = 0.0004; I^2^ = 54%). Of note, significant heterogeneity between the two subgroups was found (heterogeneity test *p* = 0.04; I^2^ = 64%) (Fig. 8).

##### Days of hospital stays

**Fig. 9:**
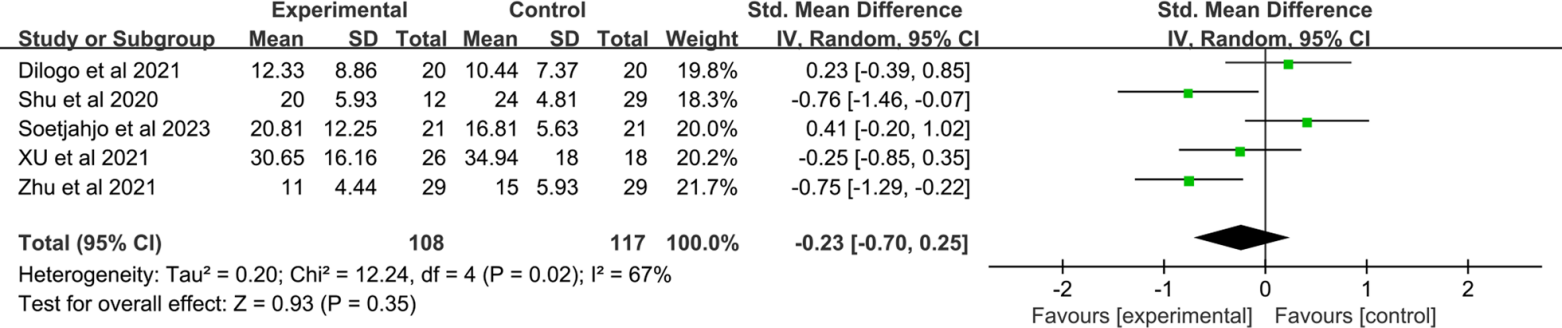
Forest plot of secondary indicators: days of hospital stays

The average length of hospital was reported in four studies of 108 patients in the MSC group and 117 patients in the control group. Significant heterogeneity was found between both groups (I^2^ = 67%; Q test *p* = 0.02). We adopted a random-effects model. The pooled analysis showed no significant difference between the MSC and control groups (WMD: − 0.23; 95% CI [− 0.70, 0.25]; *p* = 0.35) (Fig. 9).

##### The number of patients requiring respiratory support

Four studies with a total of 217 patients were enrolled. A fixed-effects meta-analysis demonstrated no substantial difference between the MSC group and control group (RR: 0.86; 95% CI [0.69, 1.07]; *p* = 0.29; I^2^ = 21%; Q test *p* = 0.17), suggesting patients treated with MSC may have better respiratory function compared to conventional therapy (Additional file1: Fig. S1A).

##### Duration of oxygen therapy

We enrolled three studies of 106 patients in the MSC group and 79 patients in the control group. Similarly, a fixed-effects model was used to reveal that no statistically significant differences were observed between the experiment and control group (WMD: − 2.31; 95% CI [− 5.79, 1.17]; *p* = 0.19; I^2^ = 0%; Q test *p* = 0.57) (Additional file1: Fig. S1B).

#### Immune biomarkers

##### CRP

**Fig. 10:**
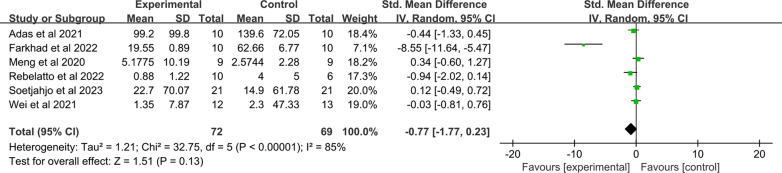
Forest plot of secondary indicators: changes in CRP in MSC and control groups

The six articles of 14 included studies included have reported the CRP levels of 141 patients with COVID-19. A meta-analysis using a random-effects model indicated that no statistically significant differences were observed in the MSC group and control group (SMD: − 0.77; 95% CI [− 1.77, 0.23]; *p* = 0.13; I^2^ = 85%; Q test *p* < 0.00001) (Fig. 10).

##### IL-6

**Fig. 11:**
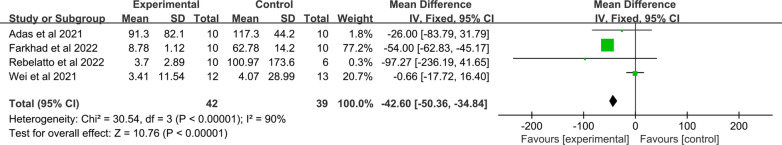
Forest plot of secondary indicators: changes in IL-6 in MSC and control groups (fixed-effects model)

The four studies reported the number of patients who had IL-6 after treatments in both the MSC and control groups. A meta-analysis with fixed-effects model showed that compared with the control group, patients in the MSC group significantly decreased levels of IL-6 (WMD: − 42.60; 95% CI [− 50.36, − 34.84]; *p* < 0.00001), but there is a significant heterogeneity (I^2^ = 90%; Q test *p* < 0.00001) (Fig. 11) Notably, when we adopted the random-effects model, no significant decrease was observed both the two groups (SMD: − 1.33; 95% CI [− 2.80, 0.13]; *p* = 0.07; I^2^ = 87%; Q test *p* < 0.0001) (Additional file1: Fig. S1D).

##### D-dimer levels

Two studies of 14 included studies have reported the levels of D-dimer. We adopted the random-effects model, no statistically significant differences were observed between the experiment and control group (SMD: 0.03; 95% CI [− 1.12, 1.18]; *p* = 0.96; I^2^ = 68%; Q test *p* = 0.08) (Additional file1: Fig. S1E).

##### Serum ferritin levels

Two studies with a total of 38 patients were enrolled. The results of a random-effects model showed that no statistically significant differences were observed (SMD: − 0.52; 95% CI [− 1.21, 0.17]; *p* = 0.14; I^2^ = 10%; Q test *p* = 0.29) (Additional file1: Fig. S1F).

##### PCT levels

The PCT was reported in three studies of 43 patients in the MSC group and 44 patients in the control group. There was no heterogeneity between the two studies (I^2^ = 0%; Q test *p* = 0.46). The fixed effects model was adopted. The pooling results reveal that no statistically significant differences were observed between the experiment and control group (WMD: − 0.03; 95% CI [− 0.12, 0.07]; *p* = 0.60) (Additional file1: Fig. S1G).

#### Sensitivity analysis

**Fig. 12:**
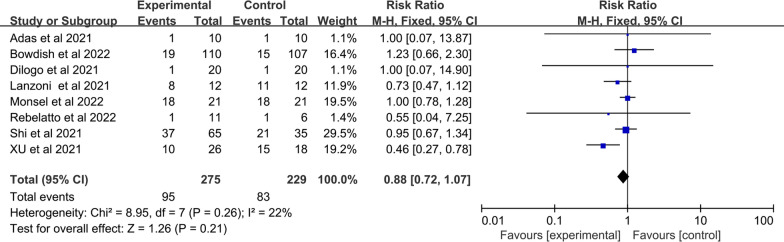
Forest plot of primary indicator: pooled results of AEs after eliminating heterogeneous

To detect sources of heterogeneity, we performed the sensitivity analysis for the outcome of significant heterogeneity. Forest plots of AEs showed heterogeneity test I^2^ = 46%, Q test *p* = 0.06. Heterogeneity in the pooled results for AEs decreased when the study of Zhu et al. was excluded. However, there was no statistically significant difference between the two groups (SMD: 0.88; 95% CI [0.72, 1.07]; *p* = 0.21; I^2^ = 22%; Q test *p* = 0.26) (Fig. 12). In addition, Forest plots of CRP indicated high heterogeneity test I^2^ = 85%; Q test *p* < 0.00001 (Fig. 10). We speculated that the inclusion of only non-severe participants in the study conducted by Farkhad et al. was a source of heterogeneity. Upon the exclusion of this study, the combined CRP data displayed lowered heterogeneity (I^2^ = 6%; Q test *p* = 0.37) (Additional file1: Fig. S1C).

#### Descriptive analysis

##### IL-10

It has long been documented that MSC infusion increases levels of anti-inflammatory factors including IL-10 [32, 43]. To be noted, Farkhad et al. documented that IL-10 was significantly increased after the 5th, 10th, and 17th days in the UC-MSC intervention group, compared to the control group [35].

##### Fibrinogen

Among the included studies, Adas et al*.* reported MSC transplantation significantly decreased fibrinogen levels (*p* < 0.05), especially after the 4th day [32].

#### Publication *bias*

**Fig. 13:**
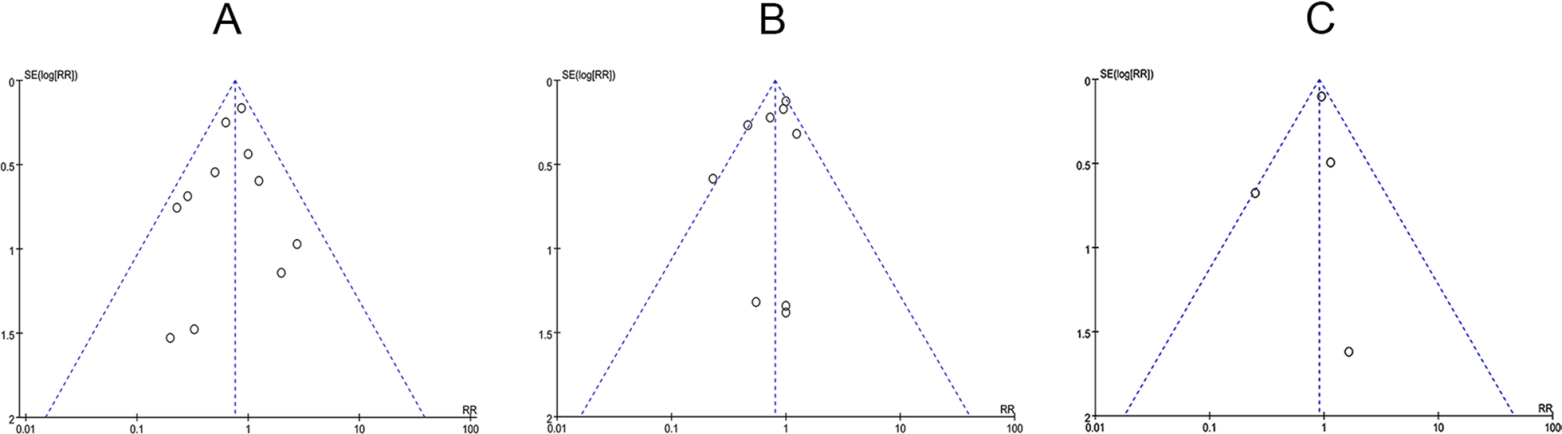
Funnel plot of publication bias: **A** Funnel plots on the mortality from eleven RCTs. **B** Funnel plots on the incidence of AEs from eight RCTs. **C** Funnel plots on the incidence of SAEs from six RCTs

We used Review Manager 5.4 to assess whether the primary indicators (such as mortality, AEs, and SAEs) were affected by publication bias. The funnel plot was applied to determine the symmetry. Based on Fig. 13, the funnel plots for the main indicators are largely symmetrical, suggesting the absence of publication bias in the pooled results for mortality, AEs, and SAEs.

## Discussion

Since the emergence of the COVID-19 pandemic, the relentless grip of COVID-19 has severely hampered global progress in both the health and economic domains [53–55]. There are currently no effective anti-SARS-CoV-2 medications, the standard treatment approach is symptomatic and pharmacological [56, 57]. Furthermore, COVID-19 patients exhibit particular immune-function abnormalities and hyperinflammation states [18, 58, 59]. The overactivated immune response is a typical clinical symptom in critically ill patients [44]. It has been shown that MSCs are capable of ameliorating inflammation stress by producing prostaglandin E2 (PGE2), heme oxygenase-1 (HO-1), and interleukin-10 (IL-10) [60]. Notably, MSCs reduce inflammation and alveolar damage in mice with acute lung injury (ALI) induced by lipopolysaccharide (LPS) [61]. In addition, partial clinical trials indicated that MSC infusion reduced mortality in COVID-19 patients without increasing the incidence of AEs [49]. Currently, we included the recently updated clinical RCTs to conduct a meta-analysis and systematically evaluate the efficacy and safety of MSC in the treatment of COVID-19. Our results suggest that MSC infusion significantly declined mortality and days to hospital stays and improved clinical survival rate. Remarkably, compared with the standard treatment group, MSC inoculation did not increase the occurrence of AEs, which suggested the safety of MSC therapy for COVID-19. In addition, further analysis of the following study might provide new perspectives for clinical research in the future.

The lower respiratory tract is the primary organ affected by COVID-19, which results in severe respiratory distress syndrome, multiple organ damage, and eventually patient death [62, 63]. Herein, mortality is one of the principal issues for evaluating the effectiveness of MSCs for COVID-19 patients. The results of the meta-analysis showed that COVID‐19 patients with MSC treatment exerted an improved patient survival rate than the control group (RR: 0.76; 95% CI [0.60, 0.96]; *p* = 0.02). Similarly, Wang et al. found that MSC infusion decreased mortality in patients with both COVID-19-induced ARDS and conventional ARDS. However, Rebelatto et al*.* found higher mortality in the MSC treatment group compared with the conventional treatment group, which highlighted the potential for baseline imbalances due to the small sample, demonstrating that the MSC group may be more vulnerable than the placebo group.

The enrolled studies reinforced that the most common causes of death in COVID-19 patients were subsequent bacterial infections, myocardial infarction and thromboembolism, multiorgan failure, or sepsis. Importantly, none of these studies reported death cases related to MSC treatment. Currently, the immunoregulatory effects of MSCs in the treatment of COVID-19 patients have been confirmed by reducing inflammation and overactive immune responses [64]. However, more studies are required to further confirm this conclusion.

Among patients with COVID-19, type I and II alveolar epithelial cells that express angiotensin-converting enzyme 2 (ACE2) are infected by SARS-CoV-2. These cells emit chemokines, which activate the neutrophils, monocytes, and T cells [65, 66]. When the immune system attempts to eradicate the virus, recruitment of abundant inflammatory cells and an aberrant cascade of inflammatory responses is onset, which in turn promotes the generation of more proinflammatory cytokines such as tumor necrosis factor-alpha (TNF-α), interferon-gamma (IFN-γ), interleukin-1 (IL-1), interleukin-6 (IL-6), interleukin-2 (IL-2), and CRP, collectively termed as CSS [67–69]. CSS is one of the leading causes of death in COVID-19 patients [70]. For example, IL-6 levels have garnered a lot of attention as a mediator of devasting inflammation [71]. Currently, four of the included eleven RCTs reported IL-6 measurements that could be extracted and pooled. The results of our meta-analysis based on the fix-effects model suggested that there was a significant difference between the MSC intervention group and the control group (*p* < 0.00001), but not in the random-effects model (*p* = 0.07). Although there was a high degree of heterogeneity, which may be related to inconsistencies in the physical condition of recruited patients between studies. Generally, MSC had a positive trend toward lowering IL-6 levels, and it still calls for further clinical studies in the future [72, 73].

On the other hand, MSC infusion also promoted the release of anti-inflammatory cytokines. The increasing level of IL-10 was observed after MSC infusion in three studies [32, 34, 43]. Consistently, Rebelatto et al*.* indicated that the MSC group significantly elevated the levels of IL-10 compared with the control group. To sum up, MSC exhibits the capacity of inhibiting the levels of pro-inflammatory cytokines and conversely increasing the secretion of anti-inflammatory [74–78]. Thus, the immune dysfunction of patients with COVID-19 was corrected, which might be the primary mechanism for MSC against COVID-19 [74, 75]. More strictly, large-cohort RCTs with a greater number of patients are required to further investigate the effects of MSCs in eliminating inflammatory cytokines.

Currently, MSC is infused through a variety of routes. MSCs may accumulate in the pulmonary microcirculatory system after intravenous transplantation, which could benefit injured alveolar epithelial cells, prevent pulmonary fibrosis, and enable MSCs to migrate to extrapulmonary organs [79–82]. Other administration routes, including subcutaneous, intratracheal, pulmonary artery, and intranasal, have also been applied for MSC infusion in treating COVID-19 patients [83]. Numerous benefits are offered by these local delivery methods, including increased application efficiency, prolonged cell half-life, and less off-target effects on other organs [83]. Notably, the consolidated information from the 14 included studies suggests consistent delivery routes, namely, intravenous infusion. Mechanistically speaking, this might facilitate endogenous repair and suppress hypercytokinemia by the regeneration function of MSCs [84]. Additionally, more researches are still needed to further assess its safety and reliability between intravenous infusion and other routes of MSC transplantation.

Concerning adverse events (AEs), we pooled the results of included studies and found that the incidence of AEs was significantly decreased in the MSC group compared with the control group (*p* = 0.03). Significant heterogeneity between studies was observed (I^2^ = 46%). To test the stability of our results, we carried out a sensitivity analysis by excluding one study [44], the following result showed that the heterogeneity was significantly reduced (I^2^ = 26%). It is worth noting that the patient characteristics which were varied between studies, such as age, and condition of illness, might be the primary reason for heterogeneity. Herein, the majority of the included studies enrolled severe or critical COVID-19 pneumonia patients, while Zhu et al. and Monsel et al. enrolled patients with mild/moderate COVID-19 pneumonia in the research. In addition, the clinical definition of AE symptoms varied across these researches. In other words, the types of AEs in these studies were diverse. As for the severe adverse events (SAEs), only six studies documented SAEs. The pooling results showed no statistically significant differences were found in the occurrence of SAEs in the MSC-treated and control groups (*p* = 0.32). Last but not least, none of the enrolled studies documented MSC infusion-related AEs or SAEs, suggesting that MSC injections are safe in treating COVID-19 patients as of now. However, it still lacks long-term follow-up research results regarding the tolerability and safety of MSC infusion. Furthermore, this conclusion should be interpreted cautiously, and more multicenter, RCTs and long-term follow-up studies are needed to determine the safety of MSC for COVID-19.

Among COVID-19 patients, the context of coagulopathy could be related to elevated D-dimer, fibrin degradation products (FDP), high serum ferritin values, and a high risk of venous thromboembolism [85–87]. The presence of coagulopathy is regarded as part of the systemic inflammatory response syndrome and is an important feature of severe COVID-19 patients [88]. The results of our meta-analysis showed there was no statistically significant difference in the levels of D-dimer and serum ferritin after the MSC therapy for patients with COVID-19. However, the sample size and external validity of the enrolled studies exert bias for the representativeness of pooled results. Consequently, further larger RCTs are required to address these problems.

Importantly, we are also concerned about the VEGF which plays a significant role in the recovery of impaired lung tissue and may be associated with vascular remodeling in COVID-19 patients [89, 90]. Adas et al. and Dilogo et al. reported that MSC infusion significantly increases VEGF levels [32, 34]. Similarly, Leng et al. also reported an increase in VEGF levels in the MSCs intervention group compared with the control group [77]. The keratinocyte growth factor, VEGF, and hepatocyte growth factor potently facilitated the post-injury repair process of alveolar epithelial cell damage [75, 91]. Thus, MSC not only acts as an immunomodulatory and anti-inflammatory mediator but also regenerates and repairs damaged lung tissue in patients with COVID-19 [92]. As demonstrated by Shu et al. MSC infusion improves Computed Tomography (CT) scores, the number of lobes involved, and the ground-glass opacity image for COVID-19 patients [41]. Consistently, the MSC group revealed a significant improvement in these clinical indicators after MSC inoculation (*p* < 0.05) [43]. However, owing to the limited number of included studies that could not be meta-analyzed, we should be biased in the interpretation of this result. More large-sample, multi-center clinical trials should be adopted to further confirm our hypothesis.

The source of MSC should also be taken into consideration. Typically, MSCs can be isolated from multiple tissues in the body, which exhibit differences in homing and differentiation ability [93–95]. A retrieval for registered trials of MSC treatment for COVID-19 was performed (*ClinicalTrials.gov*). UC-MSC is the most frequently applied cell type and is successively followed by BM, adipose tissue, dental pulp, placenta, and olfactory mucosa [96]. Presently, we included studies that applied UC-MSC as stem cell-based medications against COVID-19 in this meta-analysis. UC-MSC is inherited with advantages including quick self-renewal and proliferation, low immunogenicity, and a simpler harvesting process. Previous meta-analysis performed by McIntyre et al. indicated that the cord blood-MSC and BM-MSC showed a favorable therapeutic effect in animal models of acute lung injury [97]. Unfortunately, this result is based on small amounts of studies, and larger-sample clinical trials are required to further investigate the therapeutic effects of multiple types of MSCs against COVID-19.

Notably, MSC-derived exosomes (MSC-Exo) are emerging as a safe and effective treatment strategy for COVID-19 according to the present results of epidemiological studies [98–101]. Previous publications have also concluded that MSC-Exo is a promising cell-free therapy for COVID-19 patients [46, 102]. MSC-Exo exerts the capacity to pass the blood–brain barrier and has multiple advantages including less immunogenicity, high stability, ease of storage etc*.* [46, 103, 104] Recently, non-randomized open-label cohort research demonstrated that MSC-Exo therapy promoted immunity function and reduced cytokine storm in COVID-19 patients. In addition, the patient’s treatment experience was improved significantly after the MSC-secretome was inhaled by COVID-19 patients [105]. Importantly, more studies are still required to further investigate the therapeutic potential of MSC-Exo in treating COVID-19.

Upon including the recently updated RCTs, we assessed the efficacy and safety of MSC in the therapy of patients with COVID-19 in this meta-analysis and provided recommendations for clinical application. However, there are several limitations to this meta-analysis. Primarily, the current completed clinical trials were inadequate. As a result, we are unable to monitor and analyze the pulmonary function in patients. In addition, subgroup analyses regarding cell dose, cell type, patient severity, and route of infusion were limited by the small sample size, and can not explore potential sources of heterogeneity between studies. Furthermore, the absence of RCTs also resulted in a pooled small sample size in the meta-analysis for a few indicators, such as the levels of IL-6 and CRP. The pooled results might be biased, thus limiting the confidence of the results. Typically, the pooled results showed infusion of MSC did not increase the occurrence of AEs, statistically significant heterogeneity was observed among the included studies (I^2^ = 46%) which was reduced upon exclusion of one study conducted by Zhu et al. (I^2^ = 26%). Therefore, we should be cautious about this interpretation. Last but not least, more prospective, multi-center, randomized controlled trials with large sample sizes and long-term follow-up studies should be conducted to determine the differences involving the type of MSCs, the administration formula (including number of cycles, treatment interval, and dosage), and the route of administration.

## Conclusion

In conclusion, this meta-analysis found that MSC-based therapies are safe and effective in treating COVID-19 patients. However, it is of great importance to set up a standard treatment protocol to fully maximize the efficacy of MSC, which involves determining optimal administration routes, investigating appropriate doses of cells, and defining treatment intervals. Collectively, these might facilitate further clinical applications of MSC, and reduce the severity and lethality of patients with COVID-19. Besides, MSC-Exo has also been reported to be effective and continuous. It is to be expected that more large-scale clinical trials with MSC and MSC-Exo in the treatment of COVID-19 should be conducted, which favors the further understanding of these therapeutic approaches.

### Supplementary Information

Additional file 1: Fig. S1: Forest plot of secondary indicators: (A): the number of patients requiring respiratory support (B): duration of oxygen therapy (C): changes in CRP in MSC and control groups after eliminating heterogeneous (D): changes in IL-6 in MSC and control groups (random-effects model) (E): changes in D-dimer levels in MSC and control groups (F): changes in serum ferritin levels in MSC and control groups (G): changes in PCT levels in MSC and control groups.

Additional file 2. Detailed search strategy.

Additional file 3. PRISMA 2020 Checklist.

## Data Availability

The original data contained in this study will be accessible with the publication of this article/Supplementary Material. Further inquiries can be directed to the corresponding author.

## Abbreviations

MSCs: Mesenchymal stem cells
COVID-19: Coronavirus disease 2019
MSC-Exo: MSC-derived exosomes
BM-MSCs: Bone marrow-derived MSCs
UC-MSCs: Umbilical cord-derived MSCs
ARDS: Acute respiratory distress syndrome
CD: Crohn's disease
GVHD: Graft versus host disease
CSS: Cytokine Storm Syndrome
CRP: C-reactive protein
TNF-α: Tumor necrosis factor alpha
IFN-γ: Interferon-gamma
IL-1: Interleukin-1
IL-6: Interleukin-6
IL-2: Interleukin-2
RCTs: Randomized controlled trials
AEs: Adverse events
SAEs: Serious adverse events
PCT: Rocalcitonin
