# Supplementary material for: Efficacy and safety of mesenchymal stem cells therapy in COVID-19 patients: a systematic review and meta-analysis of randomized controlled trials
Source: J Transl Med. 2024 Jun 8;22:550. doi: 10.1186/s12967-024-05358-6 (PMC11162060; doi:10.1186/s12967-024-05358-6)
Supplement: Supplementary file 1 — Additional file 1: Fig. S1: Forest plot of secondary indicators: (A): the number of patients requiring respiratory support (B): duration of oxygen therapy (C): changes in CRP in MSC and control groups after eliminating heterogeneous (D): changes in IL-6 in MSC and control groups (random-effects model) (E): changes in D-dimer levels in MSC and control groups (F): changes in serum ferritin levels in MSC and control groups (G): changes in PCT levels in MSC and control groups. [file 12967_2024_5358_MOESM1_ESM.docx]

**Supplementary Materials**

**A**

**
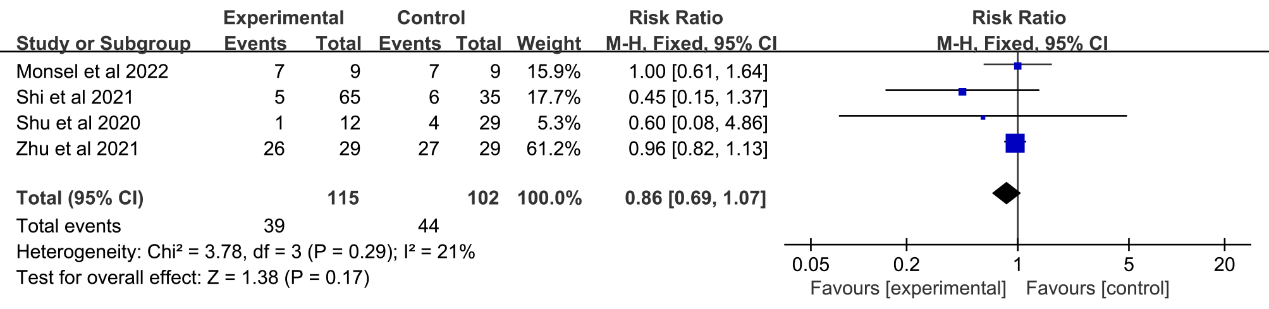
**

**B**

**
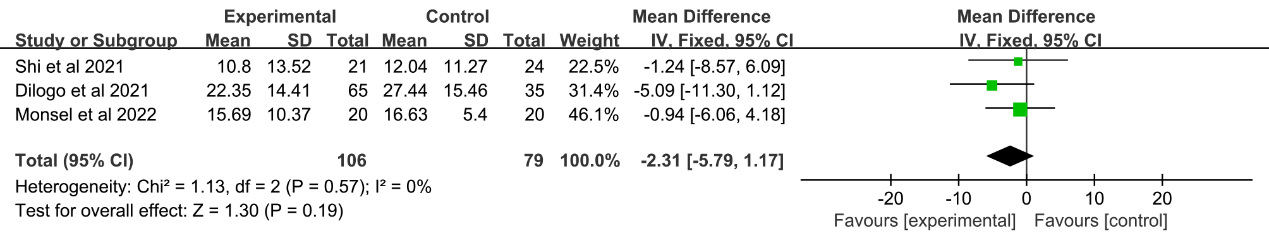
**

**C**

**
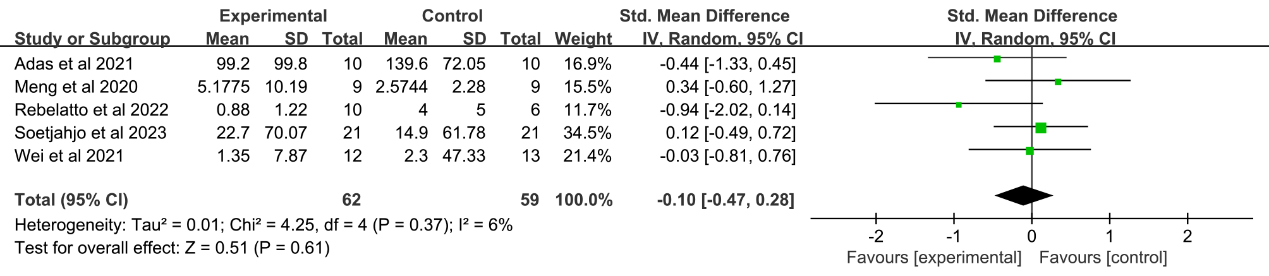
**

**D**

**
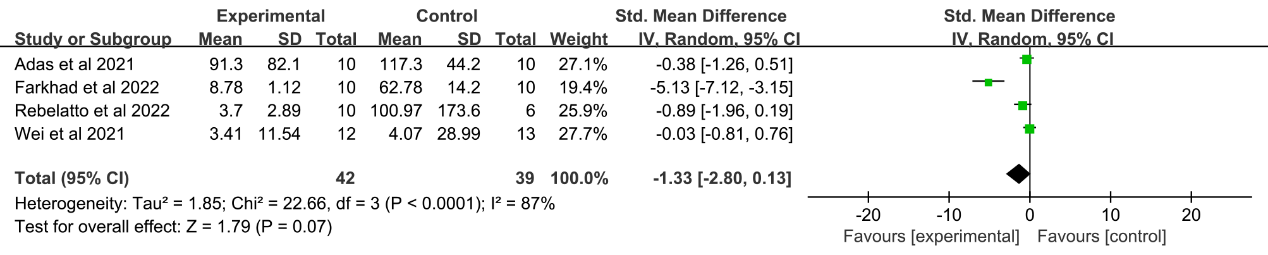
**

**E**

**
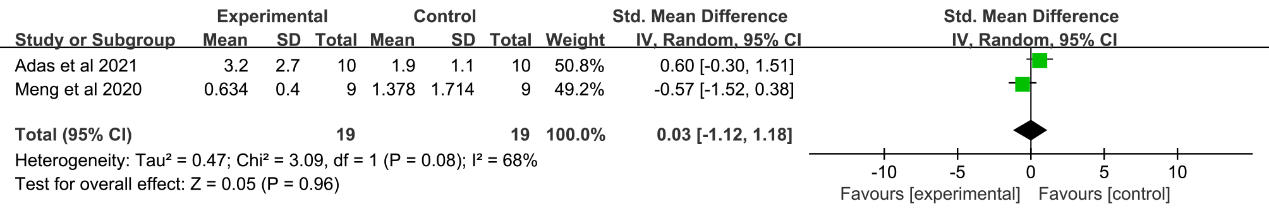
**

**F
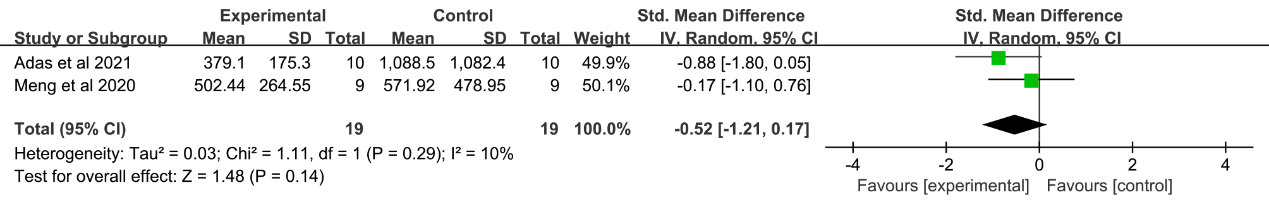
**

**G**

**
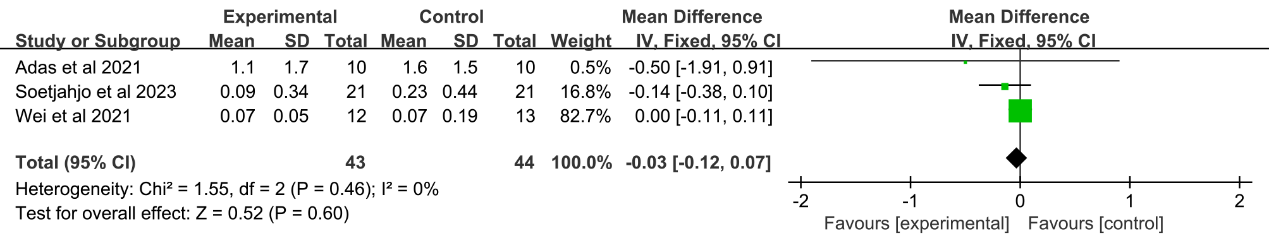
**

**Fig. S1:** Forest plot of secondary indicators: (A): the number of patients requiring respiratory support (B): duration of oxygen therapy (C): changes in CRP in MSC and control groups after eliminating heterogeneous (D): changes in IL-6 in MSC and control groups (random-effects model). (E): changes in D-dimer levels in MSC and control groups (F): changes in serum ferritin levels in MSC and control groups (G): changes in PCT levels in MSC and control groups.
